# Complications in post-bariatric body contouring surgery using a practical treatment regime to optimise the nutritional state

**DOI:** 10.1016/j.jpra.2022.06.006

**Published:** 2022-06-29

**Authors:** D.J.S. Makarawung, M. Al Nawas, H.J.M. Smelt, V.M. Monpellier, L.M. Wehmeijer, W.B. van den Berg, M.M. Hoogbergen, A.B. Mink van der Molen

**Affiliations:** a Department of Plastic, Reconstructive and Hand Surgery, St. Antonius hospital, Koekoekslaan 1, 3435 CM, Nieuwegein, the Netherlands; b Department of General Surgery, Catharina Hospital, Michelangelolaan 2, 5623 EJ, Eindhoven, the Netherlands; c Nederlandse Obesitas Kliniek (Dutch Obesity Clinic), Huis ter Heide, the Netherlands; d Department of Plastic, Reconstructive and Hand Surgery, Catharina hospital, Michelangelolaan 2, 5623 EJ, Eindhoven, the Netherlands; e Department of Plastic, Reconstructive and Hand Surgery, UMC Utrecht, Heidelberglaan 100, 3584 CX Utrecht, the Netherlands

**Keywords:** Body contouring surgery (BCS), bariatric surgery, complications, nutrition, wound healing

## Abstract

**Background:**

Post-bariatric body contouring surgery (BCS) treats redundant skin after massive weight loss; however, the complication risk is relatively high (23-70%). Most complications are wound-related, which may be partly due to a poor nutritional status after bariatric surgery. The objective of this observational study was to optimise nutrition preoperatively and assess the prevalence of wound-related complications after BCS.

**Methods:**

This prospective cohort study included 140 patients. Patients were treated according to the post-bariatric BCS guideline. Nutritional parameters were collected via pre- and peri-operative blood sampling; any deficiencies were treated. A protein-enriched diet was prescribed by a dietician 4 weeks preoperatively up until closure of all wounds. Complications were recorded using the Clavien-Dindo classification. Univariate and multivariate regression analyses were performed to identify variables associated with wound-related complications.

**Results:**

The overall wound-related complication rate was 51%. Most complications were minor, with only 4.3% was considered major. No significant differences in patient characteristics were found between patients with and without complications. Variables indicating an optimised nutritional state were not significantly associated with a decreased risk of complications; the most influential factor was a sufficient post-operative protein intake (OR 0.27, 95% CI 0.07 – 1.02, p = 0.05).

**Conclusion:**

The overall wound-related complication rate was in accordance with previous literature; however, major complications were few. This study showed a weak correlation between optimising nutritional state and better outcome after BCS, especially following a protein-enriched diet post-operatively. Therefore, we recommend continuing research on nutrition and wound-related complications, using homogeneous study populations and well-defined complications.

## Introduction

Post-bariatric body contouring surgery (BCS) has a relatively high complication rate. Studies reported complication rates of 23% to 70%, mostly wound-related.^1^, ^2^, ^3^, ^4^, ^5^, ^6^, ^7^ Several factors are associated with wound-related complications: smoking, higher age, higher pre-weight loss body mass index (BMI), more weight loss, higher current BMI, unstable weight, hypothermia, and larger weight of resected tissue.^2^^,^^4^^,^^5^^,^^7^, ^8^, ^9^, ^10^, ^11^, ^12^, ^13^ It is also suggested that patients after bariatric surgery have increased risks of complications in comparison to conservative weight loss patients, due to a higher prevalence of nutritional deficiencies caused by inadequate intake and malabsorption.^14^, ^15^, ^16^, ^17^

Nutrients play a variety of roles in wound healing and can affect wound tensile strength, collagen synthesis, and immune function, all essential elements in this process.^18^ In fact, suboptimal nutrition is associated with wound-related complications in other fields of medicine.^18^, ^19^, ^20^, ^21^, ^22^ Nutrients that may influence wound healing include: vitamin A, vitamin B, vitamin C, vitamin E, copper, iron, zinc, and protein.^18^^,^^23^, ^24^, ^25^, ^26^ Furthermore, damage to the body triggers the stress response, which results in hypermetabolism and increases glucose and protein utilisation.^27^ Therefore, nutritional support is already integrated in the treatment of burns (increased caloric intake and supplementation of vitamins A and C and copper) and malignancies.^28^^,^^29^ After bariatric surgery, patients often develop nutritional deficiencies of vitamins, minerals, and protein. Therefore, standard care includes regular assessment of dietary protein intake by a dietician and lifelong supplementation of vitamins and minerals.^30^, ^31^, ^32^ Regardless, deficiencies can still occur years after surgery.^33^ The most common deficiencies include anaemia, ferritin, folate, protein, vitamin B12, and vitamin D. Lower levels of vitamin A, vitamin B1, zinc, and copper are reported less frequently.^30^^,^^32^^,^^34^

Several studies suggested that malnutrition may be a risk factor for wound-related complications in post-bariatric BCS.^14^^,^^15^^,^^35^, ^36^, ^37^, ^38^, ^39^ Moreover, two studies reported fewer wound-related complications in patients who received protein supplementation 3 to 4 weeks preoperatively.^14^^,^^15^ However, no prospective studies have evaluated this relationship, and previous studies have lacked well-defined follow-up time and definitions of complications.

In summary, post-bariatric BCS has a high risk of wound-related complications, which might be associated with nutritional deficiencies. Our objective was to optimise protein intake and vitamin levels peri-operatively, and subsequently assess the prevalence of wound-related complications and explore variables associated with wound-related complications. We hypothesised that an optimal nutritional state would result in fewer wound-related complications post-operatively, in comparison to the existing literature. Furthermore, variables indicating optimal nutritional state were related to decreased risks of wound-related complications.

## Materials and methods

### Patient selection

This is an observational, prospective, multicentre study that was conducted in three high-volume obesity care centre in the Netherlands. Patients were included between December 2016 and October 2020. Eligible patients had a history of bariatric surgery and underwent a body contouring procedure afterwards. Previous known or suggested risk factors for complications and wound healing disturbances were exclusion criteria (Appendix 1).

In consultation with clinical epidemiologists, a minimal sample size of 120 patients was calculated. At least 30 patients with a complication were needed to evaluate factors influencing complications by logistic regression. Based on previous studies in our centre, a complication rate of 25% was assumed for the sample size calculation.^7^^,^^38^ Consequently, inclusion of 120 patients would lead to 30 patients with complications.

Ethical approval was obtained from the National Research and Ethics Committee (registration number NL53259.100.15) and the board of directors in all participating hospitals. The study was conducted in accordance with the ethical standards of the institutional and national research committee and with the 1964 Helsinki declaration and its later amendments or comparable ethical standards.

### Study procedure

**Figure 1:**
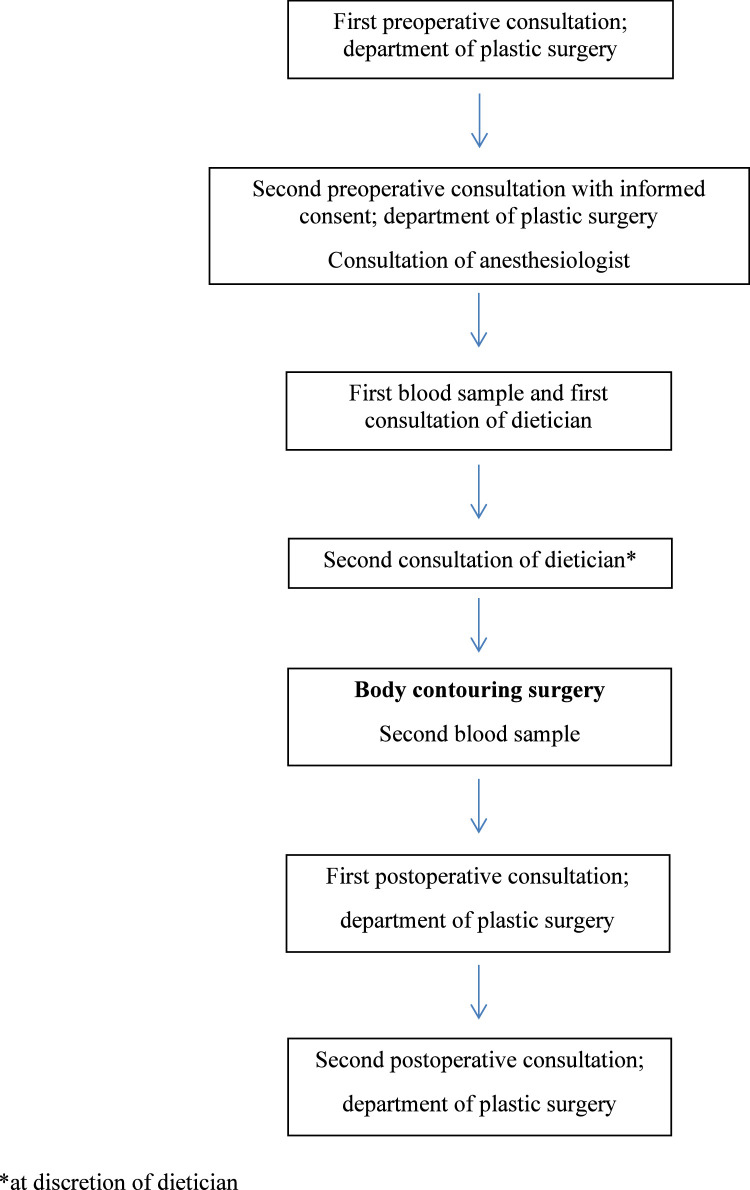
Flowchart of standard treatment according to the study protocol.

All eligible patients were informed by the plastic surgeon during their first consultation (Figure 1). Interested patients were contacted by the researcher and signed an informed consent form. Data were collected from the electronic patient record by the investigators, trained medical students, and dieticians. Data were anonymously stored using a password protected web-based application (Research Manager).

### Evaluation of nutritional status and treatment

Patients were treated according to the Clinical Practice Guideline (CPG) on Post-Bariatric Surgery of the Dutch Association of Plastic Surgery (NVPC).^40^ Based on the most common deficiencies in patients after bariatric surgery and their influence on wound healing, a preoperative assessment of albumin, ferritin, folate, haemoglobin, vitamin B12, vitamin D, and a nutritional work-up was conducted.

Approximately 8 weeks prior to BCS nutritional status was assessed by a clinical dietician. Normally, patients who had bariatric surgery require a minimum of 60 g protein per day.^41^ After consultation of experts (NUTRIM), the recommended intake was 1.5 g/kg actual body weight, based on the high prevalence of protein deficiencies after bariatric surgery and the higher requirement of protein during/after extensive surgery.^41^ If patients had a BMI > 27 kg/m^2^, the target intake was set to 1.5 g x height (m)^2^ x 27, due to the relatively higher fat free mass in persons with obesity and the relative decrease in fat free mass with increasing BMI.^42^ Patients were advised to start their protein-enriched diet 4 weeks preoperatively up to 6 weeks post-operatively or up until complete healing of all wounds. At the discretion of the dietician, a second consultation, either pre- or post-operatively, was planned.

After the first consultation, blood sampling was performed as stated in the CPG. Any deficiencies were treated according to the bariatric surgery department protocols (Appendix 2). To interpret deficiencies, the reference values of local protocols were applied, and as such, even the smallest deviation was considered a deficiency. For study purposes, a second blood sample was obtained peri-operatively to evaluate any persisting or new deficiencies. New peri-operative deficiencies were treated similarly.

Six weeks postoperatively, patients received a questionnaire to assess compliance to the treatment regime (Appendix 3).

### Standardised post-operative care

All post-operative patients were mobilised within 24 h and received prophylactic antithrombotic therapy until discharge. After abdominoplasty and lower body lift, any drains were removed when production yielded less than 50 mL per day, and all patients were advised to wear a supportive band for 3 weeks. Following brachioplasty or thigh lift procedures, any drains were removed 1 day post-operatively, and patients received compression bandages for 1 week. After reduction mammaplasty, drains were removed the first post-operative day, and patients were advised to wear a supportive bra for 6 weeks.

Two and six weeks after BCS, a standard consultation with the plastic surgeon, physician, or a trained staff nurse was planned. If case complications arose, the follow-up period was extended until all complications were treated.

### Instruments

Primary outcome variables included nutritional status, wound-related complications, and other complications arising within 30 days of surgery (Appendix 4). All interventions that treated wound-related complications were recorded. Additionally, collected variables were patient demographics, weight history, and the type of BCS. The history of bariatric surgery, use of anti-diabetic medication, and smoking status were evaluated prior to inclusion.

#### Clavien-Dindo classification

Wound-related complications and the respective interventions were graded following the Clavien-Dindo classification, which is a validated instrument for bariatric surgery that includes a therapy-orientated grading system that depends upon the therapeutic implications.^43^^,^^44^^,^^45^ Major complications were considered grade 3b or higher.

#### Questionnaire

A questionnaire assessed compliancy to the protein-enriched diet (Appendix 3). There were three possible answers: not compliant, compliant only pre- or post-operatively, and compliant during the whole study period, including the number of weeks. No validated questionnaires for assessing compliancy to diets exist; therefore, a self-developed, non-validated questionnaire was used.

### Statistical analysis

Patients’ characteristics were summarised using descriptive statistics. Normality was tested. To assess differences between patients with wound-related complications and patients without, parametric (independent T-test) or non-parametric (Mann-Whitney U test) tests were used to compare continuous variables. Chi-square tests were used for nominal variables. For study purposes, the variable BCS was transformed into three categories: 1) (fleur-de-lis) abdominoplasty, 2) (fleur-de-lis) lower body lift, and 3) mammareduction/-pexy, thigh lift procedures, brachioplasty, upper body lift, and lateral thigh lift combined with mini-abdominoplasty. A new variable ‘any deficiency’ was created for analyses. If one individual parameter of the blood sampling was missing, whereas all other parameters were within normal range, the whole blood sample was considered not deficient. If more than one variable was missing, the sample was discarded for analyses. To identify nutritional factors that influenced wound-related complications, univariate and multivariate logistic regression analysis was performed. In the multivariate model, history of smoking, location, bariatric surgery type, and BCS type were added as possible confounders. All statistical analyses were performed using the SPSS (version 25) statistical software. A two-tailed p-value < 0.05 was considered statistically significant.

## Results

### Patient demographics

A total of 158 patients were included. Of these, 17 were excluded due to incorrect inclusion (e.g., in case not all comorbidities were correctly available at the time of inclusion), and one patient decided to withdraw from the study. This resulted in 140 patients (Table 1). Patients had a mean age of 47 (SD 9.1) years, 94% were female, and 79% underwent Roux-en-Y gastric bypass. The participants had a mean percentage total weight loss (%TWL) of 40% (SD 9.9). Two patients were active smokers and quitted a minimum of four weeks preoperatively.

**Table 1:** Patient characteristics *(n = 140)*.

	Mean (SD)	Range
Gender, n (%)		
Male	8 (5.7%)	
Female	132 (94.3%)	
Age (years)	47 (9.1)	26 – 71
Body mass index (kg/m^2^)		
Pre-body contouring surgery	27.9 (3.2)	19.0 – 35.0
Pre-bariatric surgery	46.7 (5.9)	36.5 – 68.8
Total weight loss (%)	39.7 (7.9)	20.5 – 58.6
Stable weight pre-body contouring surgery (months)	18.8 (7.5)	12 – 40
History of diabetes, n (%)	11 (7.9)	
History of smoking, n (%)	18 (12.9)	
Type of bariatric surgery, n (%)		
Roux-en-Y gastric bypass	110 (78.6%)	
Sleeve gastrectomy	22 (15.7%)	
Adjustable gastric banding	8 (5.7%)	

Presented as mean (SD) unless stated otherwise.

A total of 124 patients underwent a single procedure, five patients had two serial procedures, and two patients had three serial procedures. The most frequently performed body contouring procedures were abdominoplasty (29%) and fleur-de-lis abdominoplasty (26%) (Table 2).

**Table 2:** Type of body contouring procedures.

	Number (%)
Abdominoplasty	40 (28.6%)
Fleur-de-lis abdominoplasty	36 (25.7%)
Lower body lift	5 (3.6%)
Fleur-de-lis lower body lift	21 (15.0%)
Mammoplasty/mastopexie	12 (8.6%)
Brachioplasty	8 (5.7%)
Cruroplasty	16 (11.4%)
Upper body lift	1 (0.7%)
Lateral cruroplasty and mini abdominoplasty	1 (0.7%)
**Total**	140 (100.0%)

### Nutritional status

Of the 129 patients who reported protein base intake, 93 patients (72%) achieved the minimum intake of 60 g before protein supplementation. Preoperatively, 53 patients (38%) had multiple consultations with the dietician. In 84 patients (60%), information regarding preoperative compliancy to the diet was documented by the clinical dietician. Of these, 68 patients (81%) adhered to the advised diet, with an average of 5.5 (SD 1.9) weeks before surgery. Post-operatively, 55 patients (39%) had a consultation with the dietician. In this group, sufficient protein intake was achieved by 35 patients (64%) up until an average of 4.1 (SD 1.9) weeks after surgery. A total of 108 patients (77%) completed the questionnaire that evaluated compliancy to the diet. Of these, 81 patients (75%) stated they were compliant the whole study period, while 27 patients (25%) were not.

Preoperatively, a vitamin deficiency was present in 40% of all patients, with the most frequent being vitamin D (33%) (Table 3). In 77% of these patients, vitamin D was still deficient at the time of surgery. Of all peri-operative blood samples (n = 126), 59 samples (47%) were obtained before the start of surgery and 67 (53%) after surgery. Peri-operatively, a deficiency was present in 65% of samples, with the most frequent being anaemia (42%). Most of these samples (66%) were drawn post-operatively.

**Table 3:** Pre-operative, peri-operative, and persistent pre- and peri-operative deficiencies.

Parameter	Pre-operative	Peri-operative	Pre- and peri-op
Albumin	2.3% (3/130)	17.3% (19/110)	33.3% (1/3)
Haemoglobin	9.5% (13/137)	42.1% (53/126)	69.2% (9/13)
Ferritin	9.1% (12/132)	5.3% (6/113)	20% (2/10)
Folic acid	3.8% (5/130)	10.8% (12/111)	66.7% (2/3)
Vitamin B12	3.8% (5/132)	None	None
Vitamin D	32.6% (43/132)	39.4% (41/104)	77.4% (24/31)
Any deficiency*	40.2% (53/132)	65.2% (73/112)	79.5% (35/44)

Due to missing data, results are presented as a percentage (number of deficiencies/number of available tests).

⁎ Any deficiency per procedure (yes/no).

### Wound-related complications

According to the Clavien-Dindo classification, the overall wound-related complication rate was 51%, which included grade 1: 26%, grade 2: 19%, grade 3a: 2.1%, grade 3b: 3.6%, and grade 4: 0.7% (Table 4). There was no post-operative mortality. There were four reoperations due to post-operative bleeding, of which one patient was admitted to the intensive care unit for stabilisation. One of these patients also had a later reoperation due to a wound abscess. One patient required two reoperations: first, a full thickness skin graft for a large tissue defect, and subsequent removal of the graft which had become necrotic. One patient developed a wound infection with necrosis, and a debridement was performed. The fleur-de-lis lower body lift and thigh lift procedure were most frequently associated with wound-related complications (71% and 75%, respectively) (Table 5). Mammoplasty/mastopexy was least associated with complications (8.3%).

**Table 4:** Clavien-Dindo classification of wound-related complications.

Grade		Percentage (n)
1	No pharmacological intervention, only local wound treatment	25.7% (36)
2	Pharmacological intervention	18.6% (26)
3a	Surgical, radiological, endoscopic intervention	2.1% (3)
3b	Intervention under general anesthesia	3.6% (5)
4	Life-threatening complication	0.7% (1)
5	Death of patient	None
**Total**		**50.7% (71)**

**Table 5:** Percentage of wound-related complications according to the Clavien-Dindo classification per type of procedure.

Procedure, % (n)	Grade 1	Grade 2	Grade 3a	Grade 3b	Grade 4	Total %
Abdominoplasty	35.0% (14)	10.0% (4)	2.5% (1)	2.5% (1)	None	50.0%
FdL abdominoplasty	33.3% (12)	8.3% (3)	None	5.6% (2)	2.8% (1)	50.0%
Lower body lift	40.0% (2)	None	None	None	None	40.0%
FdL lower body lift	14.3% (3)	47.6% (10)	4.8% (1)	4.8% (1)	None	71.4%
Breast surgery	None	None	None	8.3% (1)	None	8.3%
Brachioplasty	12.5% (1)	25.0% (2)	None	None	None	37.5%
Cruroplasty	25.0% (4)	43.8% (7)	6.3% (1)	None	None	75.0%
Upper body lift	None	None	None	None	None	None
Lateral cruroplasty and mini-abdominoplasty	None	None	None	None	None	None

FdL: fleur-de-lis.

### Other complications

There were 31 other complications in 23 procedures. Seven patients received a blood transfusion due to post-operative anaemia without active blood loss. Other complications were atrial fibrillation, neuropathic pain, pneumonia, and minor complications, such as obstipation, allergic reaction, and thrombophlebitis. In addition, two patients had an internal herniation related to the bariatric procedure during follow-up.

### Risk factor analysis

There were no associations between non-nutrition related variables and complications (Table 6).

**Table 6:** Univariate analysis of wound-related complications according to the Clavien-Dindo classification.

Variable	No complication (n=69)	Complication (n=71)	*P*-value
Age, years	45.9 (9.1)	48.0 (9.5)	0.18
Pre-BS BMI, kg/m^2^	46.2 (5.6)	47.2 (6.2)	0.33
Pre-BCS BMI, kg/m^2^	27.6 (3.1)	28.2 (3.3)	0.22
%TWL	39.9 (7.4)	39.5 (8.4)	0.78
Previous smoker, n (%)	6 (33.3%)	12 (66.7%)	0.15
History of diabetes^1^, n (%)	6 (54.5%)	5 (45.5%)	0.72
Weight time stable, months	18.3 (6.9)	19.2 (8.1)	0.52
Type of BS, n (%)			0.26
RYGB	54 (49.1%)	56 (50.9%)	
SG	9 (40.9%)	13 (59.1%)	
AGB	6 (75.0%)	2 (25.0%)	
Type of BCS, n (%)			0.18
Abdominoplasty	38 (50.0%)	38 (50.0%)	
Lower body lift	9 (34.6%)	17 (65.4%)	
Other	22 (57.9%)	16 (42.1%)	

Data are presented as mean (SD) unless stated otherwise.

BS: bariatric surgery; Pre-BS BMI: pre-bariatric surgery body mass index; Pre-BCS BMI: pre-body contouring surgery BMI; %TWL: percentage total weight loss; RYGB: Roux-en-Y gastric bypass; SG: sleeve gastrectomy; AGB: adjustable gastric banding.

1 Previously cured diabetes ^2^Other: mammareduction/-pexy, thigh lift, brachioplasty, upper body lift, and lateral thigh lift combined with mini-abdominoplasty

Univariate and multivariate logistic regression analysis was performed to identify nutritional variables associated with wound-related complications (Table 7). After adjusting for confounders, a preoperative sufficient diet (odds ratio (OR) 0.81; 95% CI 0.25-2.63; p = 0.72) and post-operative sufficient diet (OR 0.27; 95% CI 0.07-1.02; p = 0.05) as reported by the dietician were associated with a decreased risk of wound-related complications. Compliancy to the diet as answered by the patients (OR 0.70; 95% CI 0.26-1.90; p = 0.49) was also associated with a reduced risk. Patients who had multiple preoperative consultations with a dietician correlated with higher risks of complications (OR 1.67; 95% CI 0.78-3.59; p = 0.19). Peri-operative deficiencies of vitamins related to a reduced chance of complications (OR 0.70; 95% CI 0.28-1.75; p = 0.45).

**Table 7:** Univariate and multivariate analysis of the association of nutritional variables with wound-related complications according to the Clavien-Dindo classification.

Variable	Crude Odds Ratio [95% CI]	*P*-value	Adjusted Odds Ratio [95% CI]*	*P*-value
Base intake good^1^	0.80 [0.37 – 1.74]	0.58	0.79 [0.34 – 1.84]	0.58
Pre-operative sufficient diet^1^	0.96 [0.32 – 2.87]	0.94	0.81 [0.25 – 2.63]	0.72
Post-operative sufficient diet^1^	0.40 [0.13 – 1.25]	0.12	0.27 [0.07 – 1.02]	0.05
Compliant to protein diet^2^	0.63 [0.26 – 1.55]	0.32	0.61 [0.23 – 1.61]	0.32
Multiple consults dietician	1.23 [0.62 – 2.45]	0.55	1.67 [0.78 – 3.59]	0.19
Any deficiency pre-operatively	1.21 [0.60 – 2.43]	0.59	0.98 [0.46 – 2.14]	0.95
Any deficiency peri-operatively	1.27 [0.58 - 2.76]	0.55	0.70 [0.28 – 1.75]	0.45

1 Protein intake according to protocol as documented by dietician

2 Compliancy to the protein diet according to the questionnaire completed by patients

⁎ Adjusted for location, history of smoking, bariatric surgery type, body contouring surgery type

## Discussion

Post-bariatric BCS is associated with a relatively high wound-related complication rate. This may partly be due to nutritional deficiencies, which are common after bariatric surgery. Therefore, the aim of this study was to optimise the nutritional state of patients preoperatively and to evaluate the prevalence of wound-related complications post-operatively. Second, we explored variables associated with wound-related complications. Our study demonstrated overall wound-related complications in 51% of all patients, but only 4.3% had overall major complications. Regarding nutrition, 75% of patients followed the recommended diet. Peri-operative vitamin deficiencies were found in 65% of patients. Variables indicative of optimal nutrition had weak associations with a reduced risk of complications. However, none of these associations were statistically significant.

The overall wound-related complication rate of 51% is consistent with existing literature (23%-70%).^1^, ^2^, ^3^, ^4^, ^5^, ^6^, ^7^ Previous retrospective work performed in one of our centre found wound-related complications in 40% and 28% of patients.^7^^,^^38^ However, comparison should be made with caution due to the heterogeneity of the study populations. In these studies, more patients after gastric banding and less extensive procedures, such as scar corrections and liposuctions, were included.

The rate of major complications requiring surgical reintervention under general anaesthesia (Clavien-Dindo 3b) or admission to the intensive care unit (Clavien-Dindo 4) (4.3%) stands out positively in comparison to other studies that used the Clavien-Dindo classification (4.4% - 15%).^7^^,^^46^

Variables that indicated optimal nutrition, such as proper base intake, pre- and post-operative diet compliancy, were associated with a decreased risk of wound-related complications. Notably, a sufficient diet after surgery greatly reduced the chances of complications. Similarly, multiple dietary consults, which can be interpreted as suboptimal nutrition, were correlated to increased risks. These findings, however, were not significant, which may be a consequence of the smaller sample size due to missing data. Our results support previous work that suggested a relationship between nutrition and enhanced wound healing; however, the associations found are weak and warrant further investigation.^14^^,^^15^

Before surgery, protein intake was sufficient (>60g/day) in most patients; however, vitamin deficiencies were present in 40% of patients. Despite the standardised treatment, a high rate of these deficiencies persisted peri-operatively, especially of vitamin D. This may be due to the low compliancy with supplements in post-bariatric surgery patients.^47^ Two deficiencies stood out peri-operatively: anaemia and hypoalbuminemia. However, the reliability of these parameters as a marker for nutrition is questionable. Most blood samples with anaemia were drawn post-operatively, which could be the result of intraoperative blood loss. With regard to albumin, which can be seen as a negative acute-phase protein, reduced plasma concentrations are measured in response to tissue injury and inflammation.^48^ Consequently, albumin deficiencies may be explained by the surgery itself.^49^^,^^50^

Earlier research found risk factors such as higher age, higher pre-weight loss BMI, higher %TWL, higher BMI before BCS, and unstable weight.^2^^,^^4^^,^^5^^,^^7^, ^8^, ^9^, ^10^, ^11^, ^12^, ^13^ These variables were no risk factors for complications in our study. Exclusion criteria included a BMI > 35 kg/m^2^ and a stable weight of less than one year, which partly explains our findings. Moreover, the literature is quite variable about the relationship of abovementioned factors and complications.^5^^,^^7^^,^^8^^,^^51^

Prior work found an association between distorted wound healing and nutritional deficiencies.^14^^,^^15^^,^^39^ After bariatric surgery, deficiencies are common, and there is general consensus to treat any deficiencies and follow a protein-rich diet.^30^^,^^32^^,^^41^ Consequently, the CPG on post-bariatric surgery of the NVPC recommends a preoperative optimisation of nutrition with regard to vitamins, minerals, and protein to minimise wound-related complications.^40^ Our results suggest a weak association between an optimal nutritional state, mainly a protein-rich diet post-operatively, and a reduced risk of complications. Despite the weak association and considering the relatively low impact of the recommendations on patients, we propose to maintain the recommendation of the CPG to optimise nutritional status of patients before elective post-bariatric BCS and to conduct more research on this topic. Future studies should incorporate well-defined complications and follow-up time – like our definitions – and strive for homogeneous populations. A blood sample should be taken close to, but certainly prior to surgery, to assess any deficiencies before surgical intervention. Moreover, compliancy to the diet should be routinely assessed by a dietician using an eating journal. In these fixed conditions, predictors of complications and the effect of nutrition on wound healing should be further evaluated. Even under optimal study circumstances, it will remain challenging to establish a direct relationship of nutrition on wound healing due to the multicausality of complications.^52^

Strengths of this study were the prospective, multicentre nature, and the large number of procedures. Furthermore, the hospitals were high-volume obesity care centre with much experience in post-bariatric surgery. At last, the assessment of compliancy was a useful addition. Some limitations should be considered too. First, the ideal study design would be a randomised controlled trial. However, it was considered unethical to withhold patient's proper treatment in case of nutritional deficiencies. Therefore, in consultation with clinical epidemiologists, a prospective, observational design was conducted. Furthermore, the electronic patient file was used to assess complications, which made our results dependent of accurate documentation of the (trained) plastic surgery department staff. Moreover, we failed to properly treat all preoperative deficiencies, which led to more peri-operative deficiencies. In addition, most blood samples were obtained during or shortly after surgery, which severely hampered interpreting clinically significant differences, such as differentiating between chronic or acute anaemia. Also, there were missing blood samples and questionnaires. At last, patients with known risk factors were excluded, which could positively skew complication rates.

## Conclusion

The aim of this study was to optimise nutrition regarding vitamins, minerals, and protein and to evaluate wound-related complications in patients undergoing BCS. This study showed 51% overall wound-related complications, while only 4.3% overall major complications. Our results suggest a weak association between optimal nutritional state and reduced risk of complications. These correlations, however, did not reach statistical significance, which may be a result of our sample size and missing data. Despite the weak link, we propose to maintain the current recommendation of the Dutch CPG on post-bariatric surgery regarding optimisation of nutritional status preoperatively and advise to conduct more research. Future studies on this topic should use well-defined complications and follow-up time and strive for homogeneous populations. In these fixed conditions, the effect of nutrition on wound healing should be reassessed.

## Conflict of interest statement

“All authors declare that they have no conflict of interest”.

## Appendix Supplementary materials

Image, application 1
