# Supplementary material for: Complications in post-bariatric body contouring surgery using a practical treatment regime to optimise the nutritional state
Source: JPRAS Open. 2022 Jun 29;34:91–102. doi: 10.1016/j.jpra.2022.06.006 (PMC9535379; doi:10.1016/j.jpra.2022.06.006)
Supplement: Supplementary file 1 [file mmc1.docx]

**APPENDIX – 1**

| **Inclusion and exclusion criteria** | |
| --- | --- |
| **Inclusion** | **Exclusion** |
| 18 years of age or older | Weight loss due to dieting or exercise |
| History of bariatric surgery: | Active smoker |
| Roux-en-Y gastric bypass | Body mass index > 35 kg/m^2^ |
| Sleeve gastrectomy | Unstable weight in the past 12 months |
| Adjustable gastric banding | Diabetes (defined by using medication) |
| Body contouring surgery: | Coagulopathy |
| Abdominoplasty | Vasculitis |
| Lower or upper body lift | Connective tissue disorder |
| Brachio- or curoplasty | Kidney failure (glomerular filtration rate <30 ml/min/1,73 m^2^) |
| Mammoplasty/mastopexy | Liver failure |
|  | Use of immunosuppressive drugs |
|  | Use of anticoagulants other than acetylsalicylic acid |

**APPENDIX – 2**

| **a. Parameters, normal values, and standard treatment in *Sint Antonius Hospital*** | | |
| --- | --- | --- |
| **Parameter** | **Normal value** | **Treatment** |
| Albumin | 35-55 g/l | Assess protein intake (dietician) |
| Haemoglobin | M 7.8-10.2 mmol/l  F 7.0-9.3 mmol/l | Ferrous fumarate 200 mg 3 per day  with vitamin C for 6 months |
| Ferritin | 10-300 ug/l | Ferrous fumarate 200 mg 3 per day with vitamin C for 6 months |
| Folic acid | 10-40 nmol/l | Folic acid 0,5 mg 1 per day for 6 months |
| Vitamin B12 | 140-490 pmol/l | Hydroxocobalamin 1000 µg 1 per day for 6 months |
| Vitamin D | 50-120 nmol/l | Colecalciferol 50.000 IE/ml  1 ml every week for 6 weeks,  Than 1 ml every month for 6 moths |

| **b. Parameters, normal values, and standard treatment in *Catharina Hospital*** | | |
| --- | --- | --- |
| **Parameter** | **Normal values** | **Treatment** |
| Albumin | 30-35 g/l | Assess protein intake (dietician) |
| Hemoglobin | 7.5-10.0 mmol/l |  |
| Ferritin | 10-245 ug/l | Ferrous fumarate 200 mg 2 per day with vitamin C |
| Folic acid | >10 nmol/l | Folic acid 0.5 mg 1 per day for 6 months |
| Vitamin B12 | 140-700 pmol/l | <140: intramuscular Hydroxocobalamin 500 µg/ml, 3 doses of 1000 µg  140-300: check methylmalonic acid, when >300 nmol/l, than start treatment |
| Vitamin D | >75nmol/l | Colecalciferol 50.000 IE/ml  1 ml every week for 6 weeks,  Than 1 ml every month |

| **c. Parameters, normal values, and standard treatment in *Rijnstate Hospital*** | | |
| --- | --- | --- |
| **Parameter** | **Normal values** | **Treatment** |
| Albumin | 30-35 g/l | Assess protein intake (dietician) |
| Haemoglobin | 7.5-10.0 mmol/l | Ferrous fumarate 200 mg 2 per day  with vitamin C for 6 months |
| Ferritin | 10-245 ug/l | Ferrous fumarate 200 mg 3 per day with vitamin C |
| Folic acid | >10nmol/l | Folic acid 0.5 mg 1 per day for 6 months |
| Vitamin B12 | 140-700pmol/l | <200: intramuscular Hydroxocobalamine 500 µg/ml, 3 doses of 1000 µg |
| Vitamin D | >75nmol/l | Colecalciferol 50.000 IE/ml  1 ml every week for 6 weeks,  Then 1 ml every month |

**APPENDIX – 3**

**Post-operative questionnaire assessing compliancy to diet**

The following questions concern your consult(s) with the dietician and the recommendations you received. Please answer the questions as thoroughly as possible.

1. Have you visited the dietician once or twice?
2. Did you find the recommendations useful? If not, why not?
3. Did the recommendations include a lot of changes to your diet?
4. What did you had to change?
5. I needed to eat more
6. I needed to eat less
7. I had to change food products
8. Else, for example…..
9. Was it easy to comply with the recommendations of the dietician? If not, why not?
10. It was advised to change your diet and start 4 weeks preoperatively. How many weeks did you comply with the recommendations?
11. All 4 weeks
12. 3 – 4 weeks
13. 2 – 3 weeks
14. 0 – 1 weeks
15. Why did you not comply with the recommendations pre-operatively?
16. I found it hard to change my diet
17. I did not find it useful to change my diet
18. I forgot to change my diet
19. My weight gained
20. Else, for example…..
21. It was advised to change your diet post-operatively up until healing of all wounds. How many weeks did you comply with the recommendations?
22. The whole time
23. 4 – 5 weeks
24. 2 – 3 weeks
25. 0 – 2 weeks
26. Why did you not comply with the recommendations post-operatively?
27. I found it hard to change my diet
28. I did not find it useful to change my diet
29. I forgot to change my diet
30. My weight gained
31. Else, for example…..

**APPENDIX – 4**

| **Definition of wound-related complications** | |
| --- | --- |
| **Complication** | **Definition** |
| Hematoma | Localized collection of extravasated blood |
| Wound infection | One of the following symptoms: Pain or tenderness, localized swelling, redness/heat, purulent discharge |
| Seroma | Pocket of clear serous fluid, either clinically (physical examination or aspiration) or radiologically diagnosed |
| Wound healing disturbance | Necrosis and/or dehiscence (rupture of surgical wound along the suture line) |
| Abscess | Collection of fluid containing pus |
